# Determinants of stigma in a cohort of hellenic patients suffering from multiple sclerosis: a cross-sectional study

**DOI:** 10.1186/s12883-016-0621-4

**Published:** 2016-07-13

**Authors:** Maria Anagnostouli, Serafeim Katsavos, Artemios Artemiadis, Markos Zacharis, Paraskevi Argyrou, Ilia Theotoka, Fotini Christidi, Ioannis Zalonis, Ioannis Liappas

**Affiliations:** 1st Department of Neurology, Medical School of National and Kapodistrian University of Athens, Aeginition Hospital, Vas.Sophias ave 72-74, Athens, 115-28 Greece; 1st Department of Psychiatry, Medical School of National and Kapodistrian University of Athens, Aeginition Hospital, Vas.Sophias ave 72-74, Athens, 115-28 Greece

**Keywords:** Multiple sclerosis, Stigma, Quality of life, Stigma Scale for Chronic Illness (SSCI) Questionnaire, Socioeconomic parameters, Hellenic population

## Abstract

**Background:**

Patients suffering from several neurologic disorders may bear the “stigma” of their disease, being disqualified from full social acceptance. Although stigma is considered to be present in Multiple Sclerosis (MS), the factors that influence its levels are ambiguous. Aim of our study was to examine, for the first time in the literature, the basic determinants of stigma in a Hellenic MS-patients cohort, as well as how stigma affects their Quality-of-Life (QoL) profiles.

**Methods:**

Three hundred forty two patients were recruited in this study. Data collected concerned sociodemographic and disease-related variables, mental illness assessment, Multiple-Sclerosis-QoL-54 (MSQoL-54) and Stigma-Scale-for-Chronic-Illness-24 (SSCI-24) questionnaires. Potential determinants were evaluated with univariate statistical analyses for their contribution to total, internalized (inner-self derived) and externalized (society derived) stigma. Important findings were further evaluated on hierarchical regression models.

**Results:**

Disability levels were found to be the most powerful predictor in all stigma categories, followed by the presence of mental illness. Working and caregiving status were also ascertained as determinants of internalized stigma. Stigma levels displayed strong negative correlation with all composites of MSQoL-54.

**Conclusions:**

Stigma is present in the social environment of MS patients and was confirmed as a barrier (according to the International Classification of Functioning, Disability and Health), with detrimental effects on their QoL levels and functioning performances. Disability and mental illness were shown as the principal determinants of stigma, while financial characteristics were not as equally involved. Further validation of these results in other MS populations may provide safer conclusions, towards more efficacious patient-centered care outcomes.

## Background

Multiple Sclerosis (MS) is the most common demyelinating disease of the Central Nervous System [1]. MS patients have to cope with multiple different consequences of their disorder on their ambulation and general physical health. Inevitably, their overall perceived Quality of Life (QoL) is influenced negatively [2–4]. QoL as a concept reflects the degree in which a person’s hopes and ambitions meet his everyday experiences [5]. In medical literature, various measures of Health-Related QoL (HRQoL) are usually applied in order to determine the influence of a given disease under investigation on an individual’s perceived well-being, social interactions and overall life perception [6, 7]. HRQoL measures are unique in that they provide important, patient-derived information which is not always in accordance with the impressions of the physicians, like the information provided by the Expanded Disability Status Scale (EDSS) scores, for instance [3, 8–12]. Moreover, therapeutic success may not always be accompanied by favorable QoL outcomes and vice-versa [11, 13]. From this point of view HRQoL measures may help in actively involving MS patients in clinical decision-making [14], towards more efficacious patient-centered care outcomes.

Previous efforts to clarify the basic determining factors of QoL alterations in a variety of medical conditions led to the conclusion that the amount of stigma borne by patients plays a fundamental role. Stigmatized patients tend to seek isolation, which in turn causes decreased use of healthcare services, poor health outcomes and poor QoL [15–17]. Stigma was first defined by Goffman, in 1963 as “the situation of the individual who is disqualified from full social acceptance” [18], with an obvious consequence of social isolation – feelings of rejection. A recent revision defines stigma as “a social process, experienced or anticipated, characterized by exclusion, rejection, blame or devaluation that results from experience, perception or reasonable anticipation of an adverse social judgement about a person or group” [19]. People experiencing stigmatization to their social environment, like the ones with neurological disability, tend to acquiesce to society’s devaluation and the negative stereotypes become an accepted part of their concept of the disorder. This negative influence finally acts as a barrier, according to the International Classification of Functioning, Disability and Health (ICF) of the World Health Organization (WHO), lowering their functioning performances. Rao et al. at [20] developed the Stigma Scale for Chronic Illness (SSCI) as an instrument of measuring stigma across neurological conditions. They distinguished stigma in felt/perceived stigma (the awareness of a discriminatory stereotype in the patient’s social environment), enacted stigma (the actual experience of a discriminatory behavior), and self-stigma (internalization and acceptance of the negative stereotype, leading to shame and low self-esteem). Patients experiencing stigma are prone to increased psychological distress, like anxiety and depression [21] and they display poor health outcomes [22, 23].

The previously discussed conclusions led inevitably in several attempts of further clarification of stigma’s implications in a variety of neurological, among others, conditions. SSCI scores were found similar in people with chronic migraine and people with epilepsy, but higher than those measured in people with episodic migraine [24]. Enacted stigma in epilepsy has been repeatedly correlated negatively with QoL levels [25, 26]. Functional somatic syndromes, like chronic fatigue syndrome and fibromyalgia, were connected with greater levels of perceived stigma in comparison to a group of autoimmune disorders, with MS among them [27], which was attributed to the ambiguity in the diagnosis of the first group of situations. Finally, patients suffering from Amyotrophic Lateral Sclerosis (ALS) may be stigmatized more severely than those suffering from MS or Parkinson’s disease [28]. Nevertheless, social isolation has been reported among the basic determinants of HRQoL measures reduction in patients with Parkinson’s disease, in a previous study which was also conducted in the Hellenic population [29].

It is obvious that the aforementioned reports examined stigma in MS along with other neurological conditions, in a principal perspective of simply comparing the estimated levels. Little is known though about the basic factors which determine the stigma that MS patients encounter. Feeling stigmatized because of MS is beyond doubt, influencing the patients’ social relationships [30] and coercing them in developing manipulative behavioral strategies in order to cope [31]. From this point of view, increased comorbidity of mental illness in MS populations does not come at all as a surprise [32].

The aim of this cross-sectional study was to investigate the basic determinants of stigma in a Hellenic MS-patients cohort, as well as how stigma affects their Quality-of-Life (QoL) profiles. Besides, our study was performed during the years of economical crisis in Greece, when patients were expected to perceive the consequences of their health problems even more intensely [33, 34].

## Methods

This is a cross-sectional study that took place in the outpatient clinic (OTPC) of the 1^st^ Department of Neurology, of Medical School, of Athens National and Kapodistrian University, at Aeginition Hospital, from November 2011 to June 2014. The study protocol was approved by the Hospital’s Scientific and Ethics Committee as it was found consistent with the Declaration of Helsinki. MS patients who visited our OTPC were diagnosed as having clinically definite MS (CDMS) according to McDonald (2010) criteria [35]. Their origin was from all over Greece, since our MS Center is one of the biggest in Athens and generally in Greece. The patients were not related to each other. During the recruitment period MS patients visiting the OTPC were asked to fill in the study's questionnaires, after being fully informed about its content and the study’s goal. Patients were also asked to provide their written informed consent before answering the questionnaires. All participants that were unable to fill in the questionnaires due to serious problems in vision, hearing and/or understanding were excluded from the study. With regard to intelligence and cognition, in particular, all patients recruited should have a full intelligence quotient (FIQ of Wechsler Adult Intelligence Scale) above 79 [36], and a Mini-Mental State Examination score above 24 [37]. A medical doctor of the study’s team, along with an experienced social worker was responsible for helping and answering queries of the patients. The same medical doctor along with an experienced neurologist was responsible for gathering data related to MS. After that, each patient underwent screening by a trained clinical psychologist and evaluation by an experienced psychiatrist, for the detection of a potential mental illness. At the end of the recruitment period an independent researcher (not knowing the patients or their names) created the electronic database which served for purposes of statistical analyses.

### Measurements

1. Sociodemographical characteristics included: age, gender, marital status (married or living with others/unmarried or living alone), educational level (primary or secondary/tertiary), working status (working or student or pensioner/not working), and income per month (below 500 euros/500 or more euros).
2. Disease related characteristics: EDSS score, duration of disease in months, type of MS (relapsing-remitting/progressive), taking MS immunomodulatory drugs (yes/no), presence of at least one family member with MS (yes/no), needing a caregiver or not (yes/no) assessed by the following question: “Is there someone that you need to help you cope with your daily needs or care?”.
3. Healthy Lifestyle Score: This is a score reflecting the degree to which an individual follows a healthy daily lifestyle. Previous reports on the influence of dietary habits, exercise and smoking in MS outcomes, justified the introduction of this measure in our research [38, 39]. The score is derived by taking into account smoking, exercise and dietary habits using the following questions and scoring: 1. “Do you smoke?” Answers: No or past smoking = 2, Occasionally = 1, At least 20 cigarettes per day = 0, 2. “Do you exercise regularly i.e. at least 30 min each time?” Answers: No = 0, once or twice per week = 1, at least twice per week = 2, 3. “Please indicate the frequency that you eat each food category during a regular week” Answers: for fish/fruits/vegetables/legumes are scores as No = 0, once per week = 1, at least once per week = 2, while meat is reversely scored. Higher scores indicate healthier lifestyle (min = 0, max = 12).
4. Stigma Scale for Chronic Illness-24 (SSCI-24): This questionnaire quantifies the impact of stigma in patients with chronic illnesses [20]. The first thirteen items refer to the internalized (self) stigma (SSCI-I) asking the patient about feelings and thoughts about his/her condition (i.e. Item 7: “Because of my illness, I worried that I was a burden to others”). The next eleven items formulate the externalized (enacted) stigma subscale (SSCI-E) asking the individual about instances of actual discrimination related to the disease (i.e. Item 16: “Because of my illness, people were unkind to me”). Total score is calculated by simply adding the previous scores. Higher scores indicate higher stigma related to the chronic disease. Internal consistency of the scale in this study was found high (Cronbach’s alpha 0.921).
5. M.I.N.I. International Neuropsychiatric Interview: This is a short structured diagnostic interview, developed jointly by psychiatrists and clinicians in the United States and Europe, for DSM-IV and ICD-10 psychiatric disorders [40]. Its Hellenic version was introduced in 2004 and it has been used since then in Hellenic populations as a screening tool for psychiatric disorders [41]. The interview was undertaken by a trained clinical psychologist. MS patients were evaluated for the presence or not of at least one of the following psychiatric disorders: major depressive episode, major depressive episode and melancholy, dysthymia, suicidality, (hypo) manic episode, panic disorder, agoraphobia, social phobia, obsessive compulsive disorder, post-traumatic stress disorder, alcohol addiction/abuse, other substance addiction/abuse, psychosis, anorexia nervosa, bulimia nervosa, generalized anxiety disorder and antisocial personality disorder.
6. Multiple Sclerosis Quality of Life-54 (MSQoL-54): The MSQoL-54 is a structured, self-reported, multidimensional health-related quality of life measure [42]. This 54-item instrument generates 12 subscales: physical function, role limitations-physical, role limitations-emotional, pain, emotional well-being, energy, health perceptions, social function, cognitive function, health distress, overall quality of life, and sexual function. The summary scores are the physical health composite summary and the mental health composite summary derived from a weighted combination of scale scores. Higher scores indicate better quality of life for each composite.

### Statistical analyses

The main characteristics of our sample are presented with descriptive statistics e.g. means, standard deviations (SD), minimum and maximum values and frequencies. Each characteristic was evaluated for its contribution to stigma (SSCI-T, SSCI-I, SSCI-E) by performing univariate analyses. For interval measurements we have used Pearson’s r correlation. For categorical data we performed Mann–Whitney tests. The reason for choosing these tests is that although our sample was large, normality tests (Kolomogorov-Smirnov) and Q-Q plot inspections revealed lack of normality between groups. Each characteristic showing significant correlation with stigma was deemed as candidate determinant of stigma in the following hierarchical regression models. These models were created using a backward elimination method, after checking for models’ accuracy (e.g. influential statistics) and generalization (e.g. multicolinearity, homoscedasticity, normality and independence of residuals and linearity between independent variables and stigma). For each model adjusted R square is presented reflecting the percentage of variance of stigma explained by the models’ predictors. Level of significance was set at 0.05. Statistical analyses were done using SPSS 21.0 software (SPSS Inc., Chicago, IL, USA).

## Results

Table 1 presents the main characteristics of our sample. Missing data were low, except for income per month (35.1 %). Our sample consisted of 342 MS patients of whom the majority were females (67.5 %), with a mean age of 43.06 years, mostly married or living with someone (56.4 %) and of primary or secondary education (52 %), currently not working (79.8 %). The majority of patients reported that they had a caregiver for their daily care or needs (57.9 %), while their median EDSS score was 3.0. The mean duration of disease was 12.25 years and most patients had the relapsing-remitting type of the disease (67.6 %). Surprisingly, 30.1 % of the patients were not receiving any immunomodulatory or other main therapy for MS. 13.5 % of them reported at least one relative diagnosed with MS. The mean healthy lifestyle score was 6.96 (range: 1–12). The M.I.N.I interview and the subsequent psychiatric evaluation revealed that 36.5 % of patients had at least one psychiatric disorder. Generalized anxiety disorder was the most common (30.3 %), followed by major depressive episode (25.7 %) and dysthymia (23.4 %). Agoraphobia was present in 3.3 % of cases, while panic disorder and obsessive-compulsive disorder were present in 2.3 and 2.2 % of cases respectively. Other disorders (like alcohol abuse, substance abuse and anorexia nervosa) were present in a smaller percentage of cases.

**Table 1:** Main characteristics of the study’s sample of Multiple Sclerosis (MS) patients (*N* = 342)

Mean age ± SD (years old) (Min-Max)	43.06 ± 11.35 (17–69)	EDSS		Mean duration of disease ± SD (in months)	147.02 ± 105.37 (12–516)
		1. Median	3.0	(Min-Max)	
		(Min-Max)	(0–9.0)		
		2. Subgroups			
		0–3.5 (%)	210 (61.4)		
		4–7 (%)	117 (34.2)		
		>7 (%)	2 (0.6)		
		Missing (%)	13 (3.8)		
Gender		Caregiver		Type of MS	
Males (%)	111 (32.5)	Yes (%)	198 (57.9)	Relapsing-Remitting (%)	260 (75.8)
Females (%)	231 (67.5)	No (%)	144 (42.1)	Primary Progressive (%)	17 (5.0)
		Missing (%)	0 (0)	Secondary Progressive (%)	54 (15.7)
				Missing (%)	12 (3.5)
Marital status		Mean healthy lifestyle score ± SD	6.96 ± 2.13	Immunomodulatory drug	
Married or living with someone (%)	193 (56.4)	(Min-Max)	(1–12)	Yes (%)	227 (66.4)
Unmarried or living alone (%)	142 (41.5)			No (%)	103 (30.1)
Missing (%)	7 (2.1)			Missing (%)	12 (3.5)
Education		Presence of mental disorder		Family history of MS	
Primary or Secondary (%)	178 (52.0)	Yes (%)	125 (36.5)	Yes (%)	46 (13.5)
Tertiary (%)	155 (45.3)	No (%)	217 (63.5)	No (%)	286 (83.6)
Missing (%)	9 (2.7)	Missing (%)	0 (%)	Missing (%)	10 (2.9)
Working status		Income per month			
Working/student/pensioner (%)	60 (17.5)	<500 (%)	18 (5.3)	SD: standard deviation,	
Not Working (%)	273 (79.8)	≥500 (%)	204 (59.6)	Min: minimum, Max: maximum,	
Missing (%)	9 (2.7)	Missing (%)	120 (35.1)	EDSS: Expanded Disability Status Scale	

Table 2 presents the results of the univariate analyses of the relationships between the characteristics presented in Table 1 and stigma total and subscale scores. It is evident that with increasing age all three stigma scores also increase, reaching statistical significance (SSCI-T *p* = 0.001, SSCI-I *p* = 0.001, SSCI-E *p* = 0.006). Also, absence of work (or being a student or a pensioner) was related with higher total (*p* = 0.018) and internalized (*p* = 0.018) stigma. Similarly, an income of below 500 euros per month was significantly associated with increases of all three stigma scores (SSCI-T *p* = 0.003, SSCI-I *p* = 0.009, SSCI-E *p* = 0.013). However, due to increased missing values no further analyses was conducted with this characteristic. All stigma scores were found also significantly increased in patients needing a caregiver (SSCI-T *p* = 0.002, SSCI-I *p* = 0.004, SSCI-E *p* = 0.007), having higher EDSS scores (*p* < 0.0001 for all three stigma scores) or longer duration of MS (SSCI-T *p* = 0.001, SSCI-I *p* = 0.004, SSCI-E *p* = 0.004), suffering from progressive MS (*p* < 0.001 for all three stigma scores, all progressive forms were unified in a common category for univariate analyses, because of their small representation in our total sample, as shown in Table 1), and suffering from at least one psychiatric disorder (SSCI-T *p* < 0.001, SSCI-I *p* < 0.001, SSCI-E *p* = 0.005). Interestingly, having a positive family history for MS was associated with decreased total (*p* = 0.041) and internalized (*p* = 0.05) stigma scores.

**Table 2:** Univariate analyses examining candidate predictors of Stigma in Multiple Sclerosis (MS)

	SSCI-T	SSCI-I	SSCI-E		SSCI-T	SSCI-I	SSCI-E
Pearson’s r with age	0.19	0.19	0.16	Caregiver	(Mean values ± SD)	(Mean values ± SD)	(Mean values ± SD)
				Yes	40.67 ± 14.87	24.47 ± 10.24	16.2 ± 6.06
				No	35.78 ± 11.89	21.29 ± 8.62	14.49 ± 4.55
*p* value (Pearson’s)	**0.001**	**0.001**	**0.006**	*p* value (Mann–Whitney))	**0.002**	**0.004**	**0.007**
Gender	(Mean values ± SD)	(Mean values ± SD)	(Mean values ± SD)	Pearson’s r with Healthy Lifestyle Score	0.07	0.06	0.08
Male	37.07 ± 12.51	22.2 ± 8.43	14.87 ± 5.26				
Females	39.56 ± 14.57	23.71 ± 10.31	15.85 ± 5.70				
*p* value (Mann–Whitney)	0.17	0.39	0.09	*p* value (Pearson’s)	0.192	0.276	0.172
Marital Status	(Mean values ± SD)	(Mean values ± SD)	(Mean values ± SD)	Pearson’s r with EDSS	0.44	0.40	0.40
Married or living with someone	37.65 ± 12.79	22.33 ± 8.87	15.32 ± 5.49				
Unmarried or living alone	40.27 ± 15.36	24.45 ± 10.76	15.81 ± 5.71				
*p* value (Mann–Whitney)	0.19	0.07	0.32	*p* value (Pearson’s)	**<0.0001**	**<0.0001**	**<0.0001**
Education	(Mean values ± SD)	(Mean values ± SD)	(Mean values ± SD)	Pearson’s r with duration of disease	0.18	0.16	0.16
Primary or Secondary	39.54 ± 14.85	23.68 ± 10.18	15.86 ± 6.0				
Tertiary	37.93 ± 12.86	22.76 ± 9.25	15.18 ± 5.02				
*p* value (Mann–Whitney)	0.44	0.53	0.41	*p* value (Pearson’s)	**0.001**	**0.004**	**0.004**
Working Status	(Mean values ± SD)	(Mean values ± SD)	(Mean values ± SD)	Type of MS	(Mean values ± SD)	(Mean values ± SD)	(Mean values ± SD)
Working/student/pensioner	38.15 ± 14.19	22.82 ± 9.99	15.33 ± 5.53	RR	35.63 ± 11.44	20.97 ± 7.84	14.66 ± 4.8
Not Working	41.19 ± 12.91	24.88 ± 8.6	16.31 ± 5.73	Progressive	46.48 ± 16.25	28.69 ± 11.21	17.8 ± 6.74
*p* value (Mann–Whitney)	**0.018**	**0.018**	0.108	*p* value (Mann–Whitney)	**<0.001**	**<0.001**	**<0.001**
Income per month	(Mean values ± SD)	(Mean values ± SD)	(Mean values ± SD)	DMT Drug	(Mean values ± SD)	(Mean values ± SD)	(Mean values ± SD)
<500 (%)	47.44 ± 14.79	28.44 ± 10.03	19.0 ± 6.68	Yes	38.84 ± 13.67	23.46 ± 9.78	15.38 ± 5.19
≥500 (%)	38.24 ± 13.69	22.85 ± 9.37	15.4 ± 5.66	No	38.95 ± 14.63	22.87 ± 9.33	16.08 ± 6.5
*p* value (Mann–Whitney)	**0.003**	**0.009**	**0.013**	*p* value (Mann–Whitney)	0.897	0.648	0.909
Presence of Mental Disorder	(Mean values ± SD)	(Mean values ± SD)	(Mean values ± SD)	FH of MS	(Mean values ± SD)	(Mean values ± SD)	(Mean values ± SD)
Yes	42.58 ± 15.62	26.04 ± 10.98	16.54 ± 6.02	Yes	36.7 ± 15.31	21.81 ± 10.75	14.88 ± 5.0
No	36.45 ± 12.34	21.53 ± 8.52	14.92 ± 5.2	No	39.17 ± 13.69	23.47 ± 9.42	15.69 ± 5.7
*p* value (Mann–Whitney)	**<0.001**	**<0.001**	**0.005**	*p* value (Mann–Whitney)	**0.041**	**0.05**	0.31

*SSCI-T, -I, -E* S stigma scale for chronic illness total, internalized, externalized, *SD* standard deviation, *EDSS* expanded disability status scale, *FH* family history, *DMT* disease-modifying drug

*P* values in bold when statistical significance was reached (*p* < 0.05)

Table 3 presents the results of the hierarchical regression models for the three stigma scores. Not working, the presence of at least one mental disorder and increased EDSS were significantly associated with increased total stigma scores (*p* = 0.029, <0.0001 and <0.0001 respectively). Accordingly, the standardized beta value of EDSS (=0.42) was the most potent predictor for total stigma score. Collectively these characteristics, along with caregiver status explained 24.8 % of the overall stigma’s variance. Regarding internalized stigma, the results were similar (*p* = 0.04, <0.0001 and 0.001 respectively for the three discussed predictors) with the addition of caregiver status (*p* = 0.038) showing that MS patients needing a caregiver reported more internalized stigma. EDSS was again the strongest predictor (standardized beta of 0.28), while all the predictors presented in Table 2 explained 22.5 % of the total internalized stigma’s variance. On the other hand, backward elimination method resulted in only two statistical significant predictors of externalized stigma, the presence of mental disorder (*p* = 0.011) and EDSS (*p* < 0.0001), with the latter showing again the strongest contribution (standardized beta of 0.4). The two predictors explained 17.6 % of the total externalized stigma’s variance.

**Table 3:** Hierarchical regression models presenting predictors of Stigma in Multiple Sclerosis (MS) patients

	B (SE)	Standardized beta	*p* value	Model summary
SSCI-T				
* Constant*	26.99 (2.17)		<0.0001	F (4,304) = 26.33,
*Working (Ref.: Not working)*	−3.92 (1.79)	−0.11	**0.029**	*P* <0.001
*Caregiver (Ref.: No)*	2.75 (1.46)	0.1	**0.06**	Adjusted R square = 24.8 %
Presence of Mental Disorder (Ref: No)	5.87 (1.43)	0.2	**<0.0001**	
*EDSS*	3.42 (0.41)	0.42	**<0.0001**	
SSCI-I				
*Constant*	16.6 (1.6)		<0.0001	F (5,303) = 18.86,
*Working (Ref.: Not working)*	−2.59 (1.25)	−0.10	**0.04**	*P* <0.001
*Caregiver (Ref.: No)*	2.13 (1.02)	0.11	**0.038**	Adjusted R square = 22.5 %
Presence of Mental Disorder (Ref: No)	4.23 (1.0)	0.23	**<0.0001**	
*Type of MS (Ref.: RRMS)*	2.97 (1.61)	0.14	0.065	
*EDSS*	1.54 (0.43)	0.28	**0.001**	
SSCI-E				
*Constant*	10.77 (0.7)		<0.0001	F (2,308) = 34.1,
Presence of Mental Disorder (Ref: No)	1.53 (0.6)	0.13	**0.011**	*P* <0.001
*EDSS*	1.3 (0.17)	0.4	**<0.0001**	Adjusted R square = 17.6 %

*SSCI-T, -I, -E* S stigma scale for chronic illness total, internalized, externalized, *SE* standard error, *EDSS* expanded disability status scale

*P* values in bold when statistical significance was reached (*p* < 0.05)

Table 4 presents the correlations between stigma scores and QoL in MS patients. All three stigma scores showed strong, statistically significant negative correlations with both physical and mental health composites of the MSQoL-54 questionnaire (*p* < 0.0001 for all correlations), indicating that higher stigma was associated with worse quality of life in MS patients.

**Table 4:** Pearson’s r correlations between stigma scores and the two QoL health composites in MS patients

	Physical health (MSQOL-54)	Mental health (MSQOL-54)
SSCI-T	−0.67	−0.6
*p* value (Pearson’s)	**<0.0001**	**<0.0001**
SSCI-I	−0.67	−0.61
*p* value (Pearson’s)	**<0.0001**	**<0.0001**
SSCI-E	−0.52	−0.43
*p* value (Pearson’s)	**<0.0001**	**<0.0001**

*SSCI-T, -I, -E* S stigma scale for chronic illness total, internalized, externalized, *MSQOL* multiple sclerosis quality of life

*P* values in bold when statistical significance was reached (*p* < 0.05)

## Discussion

In this study we tried to investigate the putative predictors of stigma related to MS, adopting a stepwise approach of several sociodemographic, lifestyle and disease-related characteristics. In the first step, we checked for all possible associations by performing simple univariate analyses. In this step, we recognized several candidate significant predictors such as age, working status, income, presence of mental disorder, need for caregiver, disability status, duration of disease, type of MS and family history of MS. During the second step of hierarchical regression modeling, disability status and presence of mental disorder remained as the most potent and significant predictors of all three stigma scores (total, internalized and externalized), implying a putative strong mediating role in the relationship of the aforementioned excluded predictors with stigma. Working status and caregiving also seemed to be associated with internalized stigma, showing that unemployment (except for students or pensioners) and the need for a caregiver were associated with increased thoughts or beliefs related to MS-stigma.

Our findings concerning disability are somehow expected and in line with previous research on stigma in other neurological conditions [28]. Increased disability renders the disease more obvious to a patient’s social environment, making him more susceptible to incidents of discrimination [25, 43]. On the other hand, disability status also influenced internalized stigma, probably by facilitating thoughts of worthlessness and culpability for MS progression. After all, these results verify in part Goffman’s ideas about the ‘dramaturgical’ aspects of a disabled person’s identity management. Within this setting, stigma is not only a result of the objective impairment, but also a reflection of an individual’s ‘performance’, masking parts of its bodily functions in order to prevent social discrediting [18]. Highly disabled individuals have to cope with a continuous sense of loss of independence, further exacerbated by a potential need for a caregiver. Probably this is the reason why caregiver status retains its significance as predictor of internalized, in contrast to externalized stigma. Equally, actively working provides the necessary financial resources, which is obviously a sine-qua-non attribute for an individual in order to feel independent. Absence of occupation leads inevitably to low self-esteem, which explains the observed interference with internalized stigma. Surprisingly, despite the ongoing economical crisis in Greece, which influenced the patients of our MS sample as well (79.8 % not working), occupational status had much lower influence than disability and mental illness in total stigma formation. Its absence of significance in the regression model of external stigma determinants is an interesting fact that maybe reflects the stable Hellenic familial and social solidarity.

An effort to further analyze correlations of stigma with mental illness should take into account the reciprocal nature of connection between these two entities. First of all, mental illness in the context of MS further limits occupational opportunities, degree of acceptance from social networks and familiar structures [44]. Moreover, patients with mental illnesses seem to accept, at least in some extent, the alleged devaluating characteristics of their disorders, with obvious consequences in self-stigma [45]. On the other hand, stigma itself as a constant traumatizing experience may facilitate the emergence of psychological distress, depression in particular [21].

Other observed positive correlations with all stigma types in the univariate analyses included age, MS duration and type of disease. The loss of significance of these factors when they entered the regression models, could be explained by their relation with disability status. In other words, higher EDSS scores in higher age, longer disease duration and progressive forms are probably responsible for the noted significance. Lack of association between educational level and stigma deserves further discussion, especially if co-evaluating positive correlation with financial status. Previous researchers attributed correlation of those two factors with epileptic stigma to a probable cultural shift, due to longstanding educational efforts taking hold [24]. Perhaps, in our study group the role of educational status has given its place to family history. Indeed, Hellenic society maintains a tight family-centered structure, in contrast with other populations that were previously studied for stigma in other disorders. As a result, family quite often plays the role of educator, as well as the role of social integration promoter. In final analysis, positivity of family history for MS signifies a much easier acceptance and adjustment of a new MS case (theory that can explain our findings).

Previous findings about detrimental effects of increased stigma on QoL in several disorders were confirmed in our study, also for MS. This conclusion was clearly indicated by the significant strong negative correlations with both physical and mental health composites of the MSQoL-54 questionnaire. Although findings involved both types of stigma, the internalized type displayed slightly higher predictor qualities for both MSQoL-54 composites. In other words, MS patients had lower QoL scores mainly because of their shame and fear of discrimination, rather than actual discrimination itself. Besides, it could be hypothesized that experiencing low QoL and feeling stigmatized are not completely independent situations, but rather two interdependent aspects of the same process.

Despite the previously discussed important findings, one must consider a possible limitation in our study. Specifically, we used a convenience sample of MS patients that visited the OTPC of the Neurological Department of Athens University Hospital (although, as previously stated, their origins were for all over Greece and non-related to each other). One must consider the possibility of some patients from rural regions, who didn’t have the financial ability to reach our facilities. This fact could be responsible for a potential bias, blunting the effects of financial factors in stigma formation. Stigma experiences probably differ in relation to several sociodemographic characteristics, like race/ethnicity, even among the citizens of the same country, let alone the citizens of different countries. In other words, our results, although based on a satisfactory number of MS patients in our sample, are preliminary and any thoughts of generalization in other populations should be performed with extreme caution. Nevertheless, to our knowledge, this is the first study dedicated to the examination of determining factors of stigma in MS. Moreover, our data openly indicate, that stigma is not negligible in MS, having strong negative influence on QoL measures. Social isolation (as an indirect marker of stigma) has been previously discussed as a factor which influences negatively QoL of Hellenic patients with a neurologic condition [29]. However, observations made in Parkinson’s disease in the Hellenic population [29] are not easily transferrable to MS, because of the substantial differences in natural history and age groups that are mainly affected in these two disorders. Moreover, our study was conducted in a period of time when socioeconomic circumstances were very different.

## Conclusions

Overall, the proven existence of stigma in MS is a matter with potential serious implications that ought to be addressed. MS patients may tend towards avoiding medical care, with obvious influence in treatment outcomes. Possible interventions should include educational programs for the general public and for caregivers of MS patients, training programs for clinicians in orientation of more adequate patient-centered care services, as well as better organization of supporting and counseling structures. The detrimental effects of the two most important MS-stigma determinants, disability and mental illness, could be abated by the implementation of a prompt multi-disciplinary therapeutical approach (collaboration of neurologists, psychiatrists, psychologists and rehabilitation workers), even at the beginning of the disease. The occupational status of MS patients could be protected more adequately by improving legislation which currently regulates their occupational rights. Although, we believe that all the above proposals are in line with the scientific community’s emerging questionings, further attention should be provided in this delicate subject, in order to achieve the desirable outcomes in favor of MS patients.

## Abbreviations

ALS, amyotrophic lateral sclerosis; CDMS, clinically definite multiple sclerosis; EDSS, expanded disability status scale; FIQ, full intelligence quotient; HRQoL, health-related quality of life; ICF, International Classification of Functioning, Disability and Health; M.I.N.I, mini international neuropsychiatric interview; MS, multiple sclerosis; MSQoL-54, multiple sclerosis quality of life-54; OTPC, outpatient clinic; QoL, quality of life; SSCI, stigma scale for chronic illness; SSCI-E, stigma scale for chronic illness – externalized stigma subscale; SSCI-I, stigma scale for chronic illness – internalized stigma subscale; SSCI-T, stigma scale for chronic illness total; WHO = World Health Organization
